# Supplementary material for: The impact of assisted reproductive technology on prenatally diagnosed fetal growth restriction in dichorionic twin pregnancies
Source: PLoS One. 2020 Apr 16;15(4):e0231028. doi: 10.1371/journal.pone.0231028 (PMC7162456; doi:10.1371/journal.pone.0231028)
Supplement: S1 Table — (DOCX) [file pone.0231028.s001.docx]

S1 Table. Logistic regression analysis using gestational diabetes as the dependent variable.

|  | B | S.E. | p-value | Exp (B) | 95% CI |
| --- | --- | --- | --- | --- | --- |
| Use of ART | 0.079 | 0.242 | 0.745 | 1.082 | 0.673-1.739 |
| Nulliparity | 0.102 | 0.250 | 0.683 | 1.107 | 0.678-1.808 |
| Maternal age | 0.071 | 0.020 | 0.000 | 1.074 | 1.032-1.117 |
