# Supplementary material for: The impact of assisted reproductive technology on prenatally diagnosed fetal growth restriction in dichorionic twin pregnancies
Source: PLoS One. 2020 Apr 16;15(4):e0231028. doi: 10.1371/journal.pone.0231028 (PMC7162456; doi:10.1371/journal.pone.0231028)
Supplement: S2 Table — (DOCX) [file pone.0231028.s002.docx]

S2 Table. Logistic regression analysis using hypertensive disorders as the dependent variable.

|  | B | S.E. | p-value | Exp (B) | 95% CI |
| --- | --- | --- | --- | --- | --- |
| Use of ART | 0.243 | 0.402 | 0.545 | 1.275 | 0.580-2.805 |
| Nulliparity | 0.537 | 0.453 | 0.236 | 1.710 | 0.704-4.152 |
| Maternal age | 0.103 | 0.032 | 0.001 | 1.108 | 1.041-1.180 |
