# Supplementary material for: The impact of assisted reproductive technology on prenatally diagnosed fetal growth restriction in dichorionic twin pregnancies
Source: PLoS One. 2020 Apr 16;15(4):e0231028. doi: 10.1371/journal.pone.0231028 (PMC7162456; doi:10.1371/journal.pone.0231028)
Supplement: S3 Table — (DOCX) [file pone.0231028.s003.docx]

S3 Table. Logistic regression analysis using fetal growth restriction as the dependent variable .

|  | B | S.E. | p-value | Exp (B) | 95% CI |
| --- | --- | --- | --- | --- | --- |
| Use of ART | -0.18 | 0.235 | 0.444 | 0.836 | 0.527-1.324 |
| Nulliparity | 0.48 | 0.257 | 0.059 | 1.624 | 0.982-2.684 |
| Maternal age | -0.009 | 0.019 | 0.649 | 0.991 | 0.954-1.030 |

ART, assisted reproductive technology
