# Supplementary material for: The impact of assisted reproductive technology on prenatally diagnosed fetal growth restriction in dichorionic twin pregnancies
Source: PLoS One. 2020 Apr 16;15(4):e0231028. doi: 10.1371/journal.pone.0231028 (PMC7162456; doi:10.1371/journal.pone.0231028)
Supplement: S4 Table — (DOCX) [file pone.0231028.s004.docx]

S4 Table. Logistic regression analysis using delivery of an SGA neonate with birthweight <10^th^ percentile as the dependent variable .

|  | B | S.E. | p-value | Exp (B) | 95% CI |
| --- | --- | --- | --- | --- | --- |
| Use of ART | -0.147 | 0.162 | 0.365 | 0.863 | 0.628-1.186 |
| Nulliparity | 0.131 | 0.165 | 0.428 | 1.140 | 0.825-1.576 |
| Maternal age | 0.000 | 0.013 | 0.977 | 1.000 | 0.975-1.027 |

ART, assisted reproductive technology
