# Supplementary material for: The impact of assisted reproductive technology on prenatally diagnosed fetal growth restriction in dichorionic twin pregnancies
Source: PLoS One. 2020 Apr 16;15(4):e0231028. doi: 10.1371/journal.pone.0231028 (PMC7162456; doi:10.1371/journal.pone.0231028)
Supplement: S5 Table — (DOCX) [file pone.0231028.s005.docx]

S5 Table. Logistic regression analysis using delivery of an SGA with birthweight <5^h^ percentile as the dependent variable.

|  | B | S.E. | p-value | Exp (B) | 95% CI |
| --- | --- | --- | --- | --- | --- |
| Use of ART | -0.247 | 0.208 | 0.235 | 0.782 | 0.520-1.174 |
| Nulliparity | 0.077 | 0.209 | 0.713 | 1.080 | 0.717-1.627 |
| Maternal age | 0.008 | 0.017 | 0.640 | 1.008 | 0.975-1.042 |

ART, assisted reproductive technology
