# Supplementary material for: The impact of assisted reproductive technology on prenatally diagnosed fetal growth restriction in dichorionic twin pregnancies
Source: PLoS One. 2020 Apr 16;15(4):e0231028. doi: 10.1371/journal.pone.0231028 (PMC7162456; doi:10.1371/journal.pone.0231028)
Supplement: Attachment — Submitted filename: Response to reviewers.docx [file pone.0231028.s007.docx]

Dear Editor,

thank you for giving us the chance to further improve our manuscript. Below are all questions raised, followed by our response. We have now modified the manuscript according to the reviewers’ comments

We hope that these modifications would allow the paper to be accepted in PLOS ONE

Reviewer #1: The study have been conducted rigorously with appropriate replication and the statistical analysis has well performed.
The strength of the study is the importance of the topic, the limitation is the retrospective approach.

The authors indicate that hypertensive disorders were observed more frequently in ART group however they don’t describe preeclampsia cases especially in FGR and SGA group. R. We have now included data on the incidence of preeclampsia in the overall cohort, and in the SC and ART-group (see table 2). In addition , we have added a comment on cases of IUGR associated with preeclampsia in the result section (line 135)

More details about cesarean section category could improve the fetal outcome severity:

R. Of the 571 cesarean section performed, 59% (337/571) were elective c-sections, while the remaining 41% were emergency c-section, performed either during labor or for maternal or fetal indications in non-laboring patients. While the overall incidence of c-section was significantly higher in the IVF/ICSI group compared to the SC group, no significant difference was found in the incidence of these subcategories (elective or emergency c-section) between groups. All these results have now been included in table 2.
